# Mice carrying a schizophrenia-associated mutation of the *Arhgap10* gene are vulnerable to the effects of methamphetamine treatment on cognitive function: association with morphological abnormalities in striatal neurons

**DOI:** 10.1186/s13041-021-00735-4

**Published:** 2021-01-22

**Authors:** Kazuhiro Hada, Bolati Wulaer, Taku Nagai, Norimichi Itoh, Masahito Sawahata, Akira Sobue, Hiroyuki Mizoguchi, Daisuke Mori, Itaru Kushima, Toshitaka Nabeshima, Norio Ozaki, Kiyofumi Yamada

**Affiliations:** 1 Department of Neuropsychopharmacology and Hospital Pharmacy, Nagoya University Graduate School of Medicine, 65 Tsurumai, Showa, Nagoya, Aichi 466-8560 Japan; 2 Advanced Diagnostic System Research Laboratory, Fujita Health University Graduate School of Health Science, Aichi, 470-1192 Japan; 3 Division of Behavioral Neuropharmacology, Project Office for Neuropsychological Research Center, Fujita Health University, Aichi, 470-1192 Japan; 4 Department of Psychiatry, Nagoya University Graduate School of Medicine, Nagoya, 466-8560 Japan; 5 Japanese Drug Organization of Appropriate Use and Research, Aichi, Japan; 6 Brain and Mind Research Center, Nagoya University, Nagoya, Aichi Japan; 7 Medical Genomics Center, Nagoya University Hospital, Nagoya, 466-8560 Japan

**Keywords:** *Arhgap10*, Schizophrenia, Methamphetamine, Spine, visual discrimination

## Abstract

We recently found a significant association between exonic copy-number variations in the Rho GTPase activating protein 10 (*Arhgap10*) gene and schizophrenia in Japanese patients. Special attention was paid to one patient carrying a missense variant (p.S490P) in exon 17, which overlapped with an exonic deletion in the other allele. Accordingly, we generated a mouse model (*Arhgap10* S490P/NHEJ mice) carrying a missense variant and a coexisting frameshift mutation. We examined the spatiotemporal expression of *Arhgap10* mRNA in the brain and found the highest expression levels in the cerebellum, striatum, and nucleus accumbens (NAc), followed by the frontal cortex in adolescent mice. The expression levels of phosphorylated myosin phosphatase-targeting subunit 1 and phosphorylated p21-activated kinases in the striatum and NAc were significantly increased in *Arhgap10* S490P/NHEJ mice compared with wild-type littermates. *Arhgap10* S490P/NHEJ mice exhibited a significant increase in neuronal complexity and spine density in the striatum and NAc. There was no difference in touchscreen-based visual discrimination learning between *Arhgap10* S490P/NHEJ and wild-type mice, but a significant impairment of visual discrimination was evident in *Arhgap10* S490P/NHEJ mice but not wild-type mice when they were treated with methamphetamine. The number of c-Fos-positive cells was significantly increased after methamphetamine treatment in the dorsomedial striatum and NAc core of *Arhgap10* S490P/NHEJ mice. Taken together, these results suggested that schizophrenia-associated *Arhgap10* gene mutations result in morphological abnormality of neurons in the striatum and NAc, which may be associated with vulnerability of cognition to methamphetamine treatment.

## Introduction

Schizophrenia is a highly debilitating mental disorder that affects about 1% of the overall population. Although the pathoetiology in schizophrenia remains to be determined [1], both genetic and environmental factors are involved in developmental abnormalities in the brains of patients [2, 3]. Several environmental factors have been reported as risk factors for schizophrenia, namely maternal virus infection, malnutrition, and juvenile cannabis addiction [4–6]. Genomic studies have also suggested a role for rare copy-number variations (CNVs), defined as copy number changes including deletions and duplications of genomic regions in schizophrenia. Rare CNVs in specific loci have been identified as risk factors for schizophrenia, including deletions at 1q21.1, NRXN1, 3q29, 15q11.2, 15q13.3, and 22q11.2 and duplications at 1q21.1, 7q11.23, 15q11.2-q13.1, 16p13.1, and 16p11.2 [7–12].

We recently found a significant association between schizophrenia and exonic CNVs in the Rho GTPase activating protein 10 (*Arhgap10*) gene [13]. ARHGAP10, a member of the Rho GTPase activating proteins, inactivates RhoA and Cdc42 by converting the GTP-bound form to the GDP-bound form [14, 15]. Of note, one schizophrenia patient (Case #5) had a missense variant (p.S490P) in exon 17, which overlapped with an exonic deletion on the other allele and was located in the RhoGAP domain, leading to the activation of RhoA signaling [13]. Two mutations in the same gene, such as in Case #5, have been proposed to represent a relevant genetic model of severe schizophrenia [16]. Accordingly, we generated mice with variants of *Arhgap10* that mimicked the Case #5 genotype. The model mice, *Arhgap10* S490P/NHEJ, carrying a missense variant (p.S490P) and a coexisting frameshift mutation caused by non-homologous end-joining (NHEJ) showed an increase in anxiety-related behavior in the elevated plus-maze test and a hypersensitivity to methamphetamine in the locomotor test [13].

To elucidate the relationship between mutations in the *Arhgap10* gene and the clinical symptoms of schizophrenia, pathophysiological, morphological, and behavioral phenotypes were investigated in *Arhgap10* S490P/NHEJ mice. Previous studies indicated the morphological abnormality of pyramidal neurons in the frontal cortex of patients with schizophrenia [17–19], whereas ARHGAP10 is reported to regulate cellular morphology through the RhoA and Cdc42 pathways in fibroblasts [20]. Thus, we analyzed neuronal morphology and spine density in *Arhgap10* S490P/NHEJ mice. Moreover, schizophrenia patients exhibit cognitive dysfunction and abnormal drug reactivity: amphetamine administration at small doses can produce or enhance a psychotic reaction in patients with schizophrenia, but not in healthy controls [21, 22]. Similarly, *Arhgap10* S490P/NHEJ mice showed a higher sensitivity to methamphetamine in the locomotor test [13]. Therefore, cognitive function and vulnerability to methamphetamine in *Arhgap10* S490P/NHEJ mice were further evaluated using a touchscreen-based visual discrimination task that can evaluate higher brain functions in mice with high translational validity.

## Materials and methods

### Animals

*Arhgap10* S490P/NHEJ mutant mice (NHEJ and S490P line) were generated in a C57BL/6 J genetic background as described previously [13]. *Arhgap10* S490P/NHEJ (n = 39) mice and their wild-type littermates (n = 56) were obtained by breading two lines of heterozygous *Arhgap10* mutant mice (NHEJ line and S490P line). Male mice at 8 to 12-week-old were used in the experiment because some behavioral abnormalities have been observed in male but not female mice [13]. Mice were housed at a density of 5–6 mice per cage (28 cm length × 17 cm width × 13 cm high) in standard conditions (23 ± 1 °C, 50 ± 5% humidity) with a 12-h light/dark cycle. Food and water were available ad libitum. Animals were handled in accordance with the guidelines established by the Institutional Animal Care and Use Committee of Nagoya University, the Guiding Principles for the Care and Use of Laboratory Animals approved by the Japanese Pharmacological Society, and the National Institutes of Health Guide for the Care and Use of Laboratory Animals.

### RNA extraction and reverse transcription

Tissues were quickly dissected out on the ice, and total RNA was extracted using an RNeasy Mini Kit (Cat# 74106, QIAGEN, Hilden, Germany). Total RNA was reverse transcribed using the SuperScript III First-Strand Synthesis System (Cat# 18080-051, Invitrogen, Carlsbad, CA).

### Quantitative real-time polymerase chain reaction (qPCR)

Relative quantification was performed as described previously [23]. Absolute quantification was performed using qPCR. *Arhgap10* cDNA standard sample for absolute quantification was made from mouse brain cDNA library using specific primers for mouse *Arhgap10* (forward, GACCGTGCAGGCGTTTTCAGAAGAAGAGAG; reverse, GCTGGGACTGTCACCCTCCTCCAGCTCAAG). PCR product was inserted into a pCR-blunt II TOPO vector (Cat# K280002, Invitrogen). Plasmid DNA was cut in XhoI site and purified after electrophoresis on 2% agarose gel used to prepare the range of dilutions (10^3^–10^10^ copies/μl) to allow absolute quantification. *Arhgap10* copy number was determined from template DNA by carrying out qPCR in a total volume of 20 μl (containing 10 μl of KOD SYBR qPCR Mix (Cat# QKD-201, Toyobo Co., Ltd, Osaka, Japan), 1 μl of forward and reverse primer, 0.4 μl of 50 × ROX reference dye, 6.6 μl of DNase free water and 1 μl template DNA). The reactions were performed in ABI PRISM 7300 real-time PCR system (Thermo Fisher Scientific) using the following protocol: pre-incubation at 98 °C for 2 min (1 cycle); denaturation at 98 °C for 10 s, annealing at 60 °C for 10 s, extension at 68 °C for 1 min (repeat denaturation and extension steps for 40 cycles), melting at 95 °C for 5 s, 60 °C for 1 min, 99 °C for 15 s and 60 °C for 15 s (melt curve analysis: 1 cycle). The specificity of the primers (one PCR product amplified) was confirmed as a single melt peak. qPCR efficiency of reaction R^2^ values is more than 0.99. The primers used in the qPCR were as follows: *Arhgap10* (Cat# QT00278684, QIAGEN), *Gapdh* forward, CAATGTGTCCGTCGTGGATCT; *Gapdh* reverse, GTCCTC AGTGTAGCCCAAGATG. Raw data was described in Additional file 1: Table S2 (https://figshare.com/s/71bb6254308a3c0fb8a4).

### In situ hybridization

The probe for in situ hybridization analysis was designed from positions 1216–1571 of the *Arhgap10* genomic DNA (GenBank accession number: NM_030113.2). Dissected mouse brain at the age of 8-week old was fixed with G-Fix (Cat# STF-02, Genostaff, Tokyo, Japan) and embedded in paraffin on CT-Pro20 (Genostaff) using G-Nox (Cat# GN04, Genostaff). in situ hybridization was performed using an in situ hybridization Reagent Kit (Cat# SRK-02, Genostaff) in accordance with the manufacturer's instructions. Briefly, de-paraffinized and rehydrated brain slices (8 μm) were fixed with 10% neutral buffered formalin for 15 min at room temperature followed by treatment with proteinase K (Cat# 16414004, 4 mg/ml; Wako Pure Chemical Industries, Ltd, Osaka, Japan) in phosphate-buffered saline (PBS) for 10 min at 37 °C. After washing in PBS, samples were re-fixed with 10% neutral buffered formalin for 15 min at room temperature and placed in 0.2 M HCl for 10 min at room temperature. The PBS-washed sample was placed in a coplin jar containing 1 × G-Wash (Cat# SHW-01, Genostaff). Hybridization was performed with probes at concentrations of 300 ng/ml in G-Hybo-L/G-Hybo (Cat# RPD-02 and RPD-01, respectively; Genostaff) for 16 h at 60 °C. After hybridization, the sections were washed in 1 × G-Wash for 10 min at 60 °C, and 50% formamide in 1 × G-Wash for 10 min at 60 °C. Then, the sections were washed twice in 1 × G-Wash for 10 min at 60 °C, and twice in 0.1 × G-Wash for 10 min at 60 °C, and twice in 0.1% Tween 20 in Tris-buffered saline at room temperature. After treatment with 1 × G-Block (Cat# GB-01, Genostaff) for 15 min at room temperature, the sections were incubated with anti-digoxigenin alkaline phosphatase conjugate (Cat# 11093274910, Roche, Diagnostics GmbH, Mannheim, Germany) diluted 1:2000 with G-Block in 0.1% Tween 20 in tris-buffered saline for 1 h at room temperature. The sections were incubated in 100 mM NaCl, 50 mM MgCl_2_, 0.1% Tween 20, 100 mM Tris–HCl, pH 9.5. Coloring reactions were performed with 5-bromo-4-chloro-3-indolyl-phosphate/nitroblue tetrazolium solution (Cat# B6149, Cat# 34035, respectively; Sigma-Aldrich, MO, USA) overnight and then washed in PBS. The sections were counterstained with Kernechtrot stain solution (Cat# 40871, Muto Pure Chemicals, Tokyo, Japan), and mounted with G-Mount (Cat# GM-01, Genostaff).

### Western blotting

Western blotting was performed as described previously [24]. In brief, the striatum and NAc tissue were dissected out from 8-week-old mouse brain according to the mouse brain atlas (Franklin and Paxinos, 1997), using 2 mm diameter Biopsy punches (Cat# 52-004620, Integra Miltex, Davies Dr, York, PA, USA). The tissue was then sonicated and boiled at 99 °C in lysis buffer [complete protease inhibitor cocktail (Cat# 11873580001, Roche) for 10 min, and phosSTOP phosphatase inhibitors (Cat# 4906837001, Roche) in 1% SDS solution]. Samples were centrifuged at 15,000 rpm for 20 min. The protein concentration was determined using a DC Protein Assay Kit (Cat# 5000111JA, Bio-Rad Laboratories, Hercules, CA), and protein was boiled in sample buffer (0.5 M Tris–HCl, pH 6.8, 1% SDS, 30% glycerol, 0.0012% bromophenol blue, and 0.93% DTT); applied to an 8% SDS–polyacrylamide gel; and subsequently transferred to a polyvinylidene difluoride membrane (Cat# IPVH00010, Millipore, Darmstadt, Germany). The membranes were blocked with Blocking One-P (Cat# 05999-84, Nacalai Tesque, Kyoto, Japan) at room temperature for 1 h and incubated with a primary antibody at 4 °C overnight. Then, membranes were washed three times every 10 min with 1% Tween 20 in tris-buffered saline. After incubation with horseradish peroxidase-conjugated secondary antibody at room temperature for 1 h, the immune complex was detected using ECL Plus Western blotting detection reagents (Cat# RPN2236, GE Healthcare, Pittsburg, PA, USA). The intensities of the bands on the membranes were analyzed using a LuminoGraph I (Atto Instruments, Tokyo, Japan). To calculate the relative amount of phosphorylated proteins compared with total proteins, the same membranes were stripped with WB Stripping Solution Strong (Cat# 05677-65, Nacalai Tesque) at room temperature for 1 h and treated as described above. Because there was no change in the levels of total proteins, values of phosphorylation were normalized to the values of total proteins. All the data from western blotting were expressed as relative fold change in expression relative to the control. The primary and secondary antibodies were diluted with Can Get Signal Solution 1 and 2 (Cat# NKB-101, Toyobo), respectively, to enhance antibody-antigen binding. The primary antibodies were used as follows: a rabbit anti-myosin phosphatase-targeting subunit 1 (MYPT1) (Cat# 2634, RRID:AB_915965, 1:1,000; Cell Signaling Technology, Danvers, MA), and rabbit anti-phospho-MYPT1 (Thr696) (Cat# ABS45, RRID:AB_10562238, 1:1,000; Millipore), rabbit anti-p21-activated kinase (PAK) 1/2/3 (Cat# 2604, RRID:AB_2160225, 1:500; Cell Signaling Technology), rabbit anti-phospho-PAK1 (Ser144) /PAK2 (Ser141) (Cat# 2606, RRID:AB_2299279, 1:1,000; Cell Signaling Technology). The secondary antibodies were horseradish peroxidase-conjugated anti-rabbit IgG antibody (Cat# NA9340, RRID:AB_772191, 1:10,000; GE Healthcare). Raw data was described in Additional file 1: Table S2 (https://figshare.com/s/71bb6254308a3c0fb8a4).

### In vivo microdialysis

In vivo dialysis was performed as described previously [25]. Mice at 8-week-old were anesthetized with tribromoethanol intraperitoneal administration (i.p.; 250 mg/kg) and a guide cannula (AG-6; Cat# 801224, Eicom Corp., Kyoto, Japan) was implanted in the NAc (+ 1.5 mm anteroposterior, + 0.8 mm mediolateral from the bregma, and 4.0 mm dorsoventral from the skull) according to the mouse brain atlas (Franklin and Paxinos, 1997). Two days after recovery from surgery, a dialysis probe (A-I-6-01; Cat# 801026, membrane length of 1 mm, Eicom Corp.) was inserted through the guide cannula and perfused with artificial cerebrospinal fluid (147 mM NaCl, 4 mM KCl, and 2.3 mM CaCl_2_) at a flow rate of 1.0 μl/min. Outflow fractions were collected every 5 min. After the collection of baseline fractions, mice were treated with methamphetamine (1.0 mg/kg, i.p.). Dopamine (DA) levels in the dialysates were analyzed using a high-performance liquid chromatography system (HTEC-500, Eicom Corp.) equipped with an electrochemical detector. Raw data was described in Additional file 1: Table S2 (https://figshare.com/s/71bb6254308a3c0fb8a4).

### c-Fos immunohistochemistry

The details of the procedure were described in a previous report [26]. Briefly, animals at 8-weel-old were deeply anesthetized with tribromoethanol (250 mg/kg, i.p.) and perfused transcardially with 0.1 M phosphate buffer followed by 4% paraformaldehyde solution. The brain was removed and fixed overnight in 4% paraformaldehyde and stored in 30% sucrose, embedded in Tissue-Tek O.C.T. compound (Cat# 4583, Sakura Finetech, Tokyo, Japan), and then stored at -80 ˚C. Brain slices (30 µm thickness) were washed with PBS containing 0.3% Triton X-100 and blocked at room temperature for 2 h in a presence of 2% normal goat serum (Cat# S-1000, Vector Laboratories Inc, Burlingame CA, USA). The samples were incubated with rabbit anti-c-Fos antibody (Cat# PC38, RRID:AB_2106755, 1:10,000; EMD Millipore, Billerica, Massachusetts, USA) at 4 °C overnight. After washing with PBS, the sections were incubated with a biotinylated goat anti-rabbit IgG secondary antibody (Cat# BA-1000, RRID:AB_2313606, 1:1,000, Vector Laboratories Inc) at room temperature for 2 h. The sections were then incubated with PBS containing 0.3% hydrogen peroxide for 30 min, and treated with avidin-biotinylated horseradish peroxidase complex (Vectastain ABC kit; Cat# PK-6100, Vector Laboratories Inc) at room temperature for 2 h. Signals were visualized using the diaminobenzidine-nickel method. Only cells that had significant, above background levels of DAB staining in their nuclei were counted (diameter; 3–11 μm). The acquisition parameters were kept the same for all images. The number of c-Fos-positive cells was counted within an area of 340 × 460 µm using Metamorph software (Molecular Devices, Sunnyvale, CA, USA). Brain areas were determined according to the mouse brain atlas (Franklin and Paxinos, 1997). Nine regions of interest from three mice were used in each group. Raw data was described in Additional file 1: Table S2 (https://figshare.com/s/71bb6254308a3c0fb8a4).

### Golgi staining and morphological analysis

Golgi staining was performed using the FD Rapid Golgi Stain Kit (Cat# PK401, FD NeuroTechnologies, Ellicott City, MD) as described previously [23]. Neurolucida software (MicroBrightField Bioscience, Williston, VT) was used to trace neurons on BZ9000 bright-field microscopic (KEYENCE, Osaka, Japan) images (at 100 × magnification). Neurons were analyzed in the striatum and NAc. All of the neurons analyzed were well stained and isolated and had intact dendritic arbors. Node numbers, intersections, dendritic length, and ending numbers of each traced neuron were analyzed using NeuroExplorer software (MicroBrightField Inc). Four mice were used in each group. Two to three neurons were traced per mouse.

Dendritic spines were counted in secondary dendrites of neurons on branches. According to their morphology in previous reports [27], protrusions were distinguished into four categories; (1) mushroom spines with a large head (width > 0.6 μm); (2) stubby spines with a head but without a neck; (3) thin spines with a long neck (length > 1 μm) and small heads; and (4) branch spines with more than two heads. The number of spines was measured from 4 different secondary dendrites per mouse. Four mice were used in each group. Raw data was described in Additional file 1: Table S2 (https://figshare.com/s/71bb6254308a3c0fb8a4).

### Touchscreen-based visual discrimination task

The protocol was described in previous reports [26, 28]. Briefly, mice at 8-week-old were restricted in their access to food and water for 2 h (5–7 pm) on a day at least 1 week before the pretraining to provide enough motivation to perform the task. The food and water restriction was continued until the end of the task. The task started with 5 pretraining stages (habituation, initial touch, must touch, must initiate, and punish incorrect) to shape screen-touching behavior in mice. After mice completed this pretraining (> 75% for 2 consecutive days), they were subsequently moved to the visual discrimination task, in which trial initiation was triggered by mice touching the nozzle, and 2 stimuli (marble and fan) were then presented simultaneously in the 2 response windows. Touching the correct window resulted in the delivery of a liquid reward (20 μl). When the incorrect window was touched, the stimuli offset immediately and a 5 s time-out period was started. After an inter-trial interval (20 s), a correction trial was given instead of a new trial. In the correction trial, the same stimulus set was repeatedly presented in the same location until the mouse made a correct response. Stimulus contingencies were counterbalanced. The session finished after 1 h or 30 trials were completed, whichever came first. The total number of trials, total number of correction trials, response latencies, percentage of trials completed, and correction errors, as well as the percentage of correct responses, were analyzed.

When mice could perform with more than 80% correct responses for 2 consecutive days, the reversal learning task was begun, this stage was similar to the initial acquisition of the visual discrimination task, except that the contingency of the stimulus pair was reversed. Raw data was described in Additional file 1: Table S2 (https://figshare.com/s/71bb6254308a3c0fb8a4).

### Drug treatment

Methamphetamine (Sumitomo Dainippon Pharma, Osaka, Japan) or saline was administered i.p. to mice 2 h before immunohistochemistry. In behavioral analyses, mice were treated with methamphetamine (0.1 ml/10 g body weight) 30 min before the start of the visual discrimination test.

### Experimental setup

In the visual discrimination task, all mice were sequentially used for the normal visual discrimination learning, reversal learning, and then methamphetamine treatment. In other experiments, mice were used only once without reuse (in vivo microdialysis, c-Fos staining, Golgi staining, Western blotting, qPCR, in situ hybridization).

### Statistical analyses

All data are expressed as means ± SEM. Statistical significance was determined using Mann–Whitney U-test for comparisons of two groups. For multiple comparisons, statistical significance was determined using an analysis of variance (ANOVA) with repeated measures, and Tukey–Kramer test was used for post-hoc analysis when *F* ratios were significant. Results of statistical analyses were provided in Additional file 1: Table S1.

## Results

### Spatiotemporal expression of *Arhgap10* mRNA in the mouse brain

**Fig. 1:**
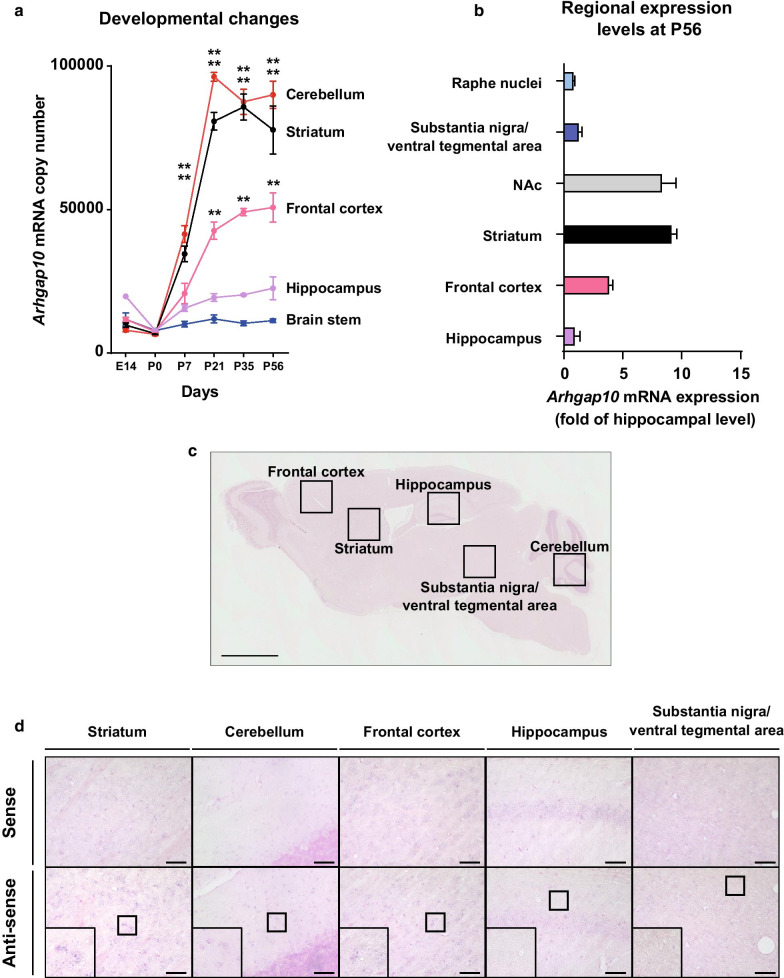
Spatiotemporal expression of *Arhgap10* in the mouse brain. **a** Temporal changes in brain *Arhgap10* mRNA levels in C57BL/6 J mice (n = 3 mice in each time point). **b** Relative expression of *Arhgap10* mRNA levels in the hippocampus, frontal cortex, striatum, nucleus accumbens (NAc), substantia nigra/ventral tegmental area, and raphe nuclei of C57BL/6 J mice at P56 (n = 3 mice). **c** Representative images showing *Arhgap10* mRNA expression at P56 by in situ hybridization. Scale bar indicates 1 mm. **d** Higher magnification of *Arhgap10* mRNA signals in the striatum, cerebellum, frontal cortex, hippocampus, and substantia nigra/ventral tegmental area of the mouse brain. Scale bar indicates 50 μm. ** p < 0.01 compare to respective expression level at P0. All data are expressed as means ± SEM

ARHGAP10 is expressed in the brain [15, 20], and it plays an important role in the development of the brain [13, 29]. However, because the spatiotemporal expression remains unknown, we first investigated the developmental changes in the levels of *Arhgap10* mRNA expression in the striatum, frontal cortex, hippocampus, cerebellum, and the brain stem (including substantia nigra, raphe nuclei, and ventral tegmental area) of naïve C57BL/6 J mice from embryonic day (E) 14 to postnatal day (P) 56, by qualifying the absolute levels of *Arhgap10* mRNA. The *Arhgap10* mRNA levels in the cerebellum, striatum, and frontal cortex increased in an age-dependent manner, and the significant increase was detected at P56 compared with the respective basal level at P0 (Fig. 1a). At P56, the highest expression levels of *Arhgap10* mRNA were observed in the striatum and NAc, followed by the frontal cortex. The expression levels in other brain regions such as the hippocampus, substantia nigra/ventral tegmental area, and raphe nuclei were low (Fig. 1b). The signal of *Arhgap10* mRNA was also detected in the striatum, frontal cortex, and cerebellum by in situ hybridization (Fig. 1c, d). Taken together, *Arhgap10* mRNA expression increased in an age-dependent manner in the mouse brain, in which the cerebellum, striatum, and NAc exhibited the highest expression levels in measured brain regions during adolescence.

### Activation of MYPT1 and PAKs in the striatum and NAc of *Arhgap10* S490P/NHEJ mice

**Fig. 2:**
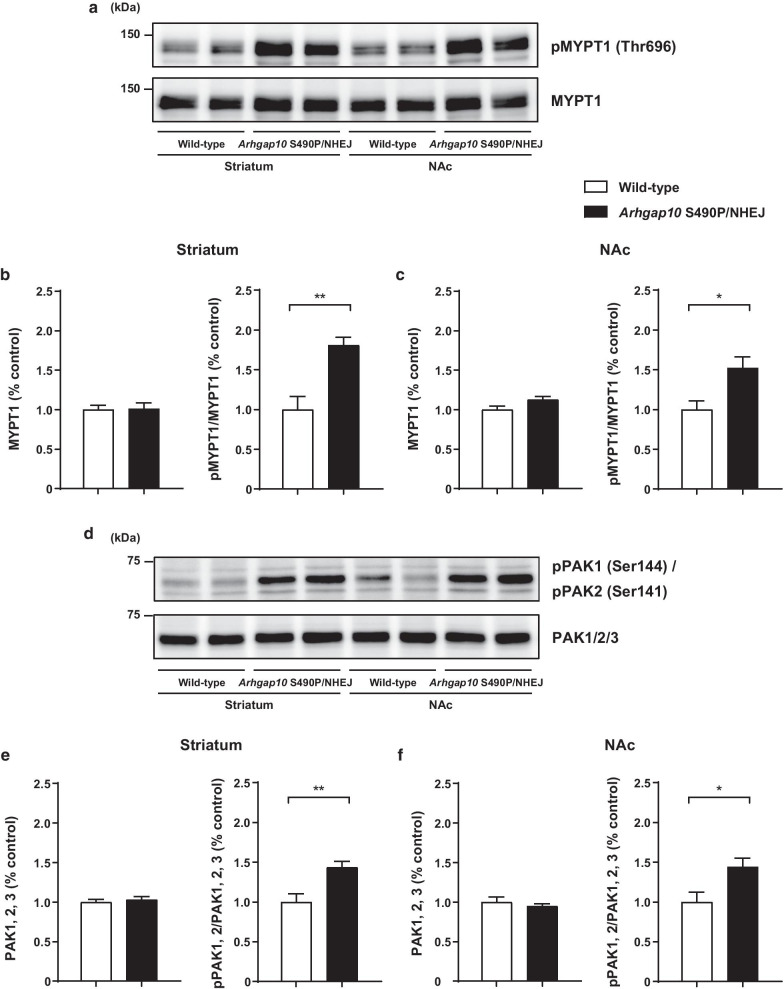
Activation of MYPT1 and PAKs in the striatum and NAc of *Arhgap10* S490P/NHEJ mice. **a**, **d** Representative endogenous protein levels of phosphorylated (upper) and total band (lower) of MYPT1 (**a**) and PAK1/2 (**d**) in the striatum and NAc. **b**, **c**, **e**, **f** Quantification of phosphorylated band intensity in the striatum and NAc was normalized to total (**b**, **e**) MYPT1 and (**c**, **f**) PAK1/2 band, respectively. *p < 0.05, **p < 0.01. All data are expressed as means ± SEM (MYPT1; n = 8 mice, PAK1/2; n = 8 mice in each genotype)

Rho-kinase is a downstream effector of RhoA [30], whereas PAKs act as the downstream effectors for Cdc42 and Rac1 [31]. Rho-kinase phosphorylates MYPT1 at Thr696, resulting in a decrease in myosin light chain phosphatase activity and an increase in the phosphorylated myosin light chain [30]. Accordingly, the effect of *Arhgap10* gene mutations on the downstream signal activity was investigated using phospho-MYPT1 and phospho-PAK1/2 levels in the striatum and NAc of *Arhgap10* S490P/NHEJ mice. The phospho-MYPT1 levels were increased in the striatum and NAc of *Arhgap10* S490P/NHEJ mice compared with wild-type littermates (Fig. 2a–c). The phospho-PAK1/2 (Ser144/141) levels were also higher in the striatum and NAc of *Arhgap10* S490P/NHEJ mice than those in wild-type littermates (Fig. 2d–f). These results suggested that *Arhgap10* S490P/NHEJ mice have an abnormality in the Rho signaling pathway in the striatum and NAc.

### Abnormal neuronal morphology in the striatum and NAc of *Arhgap10* S490P/NHEJ mice

**Fig. 3:**
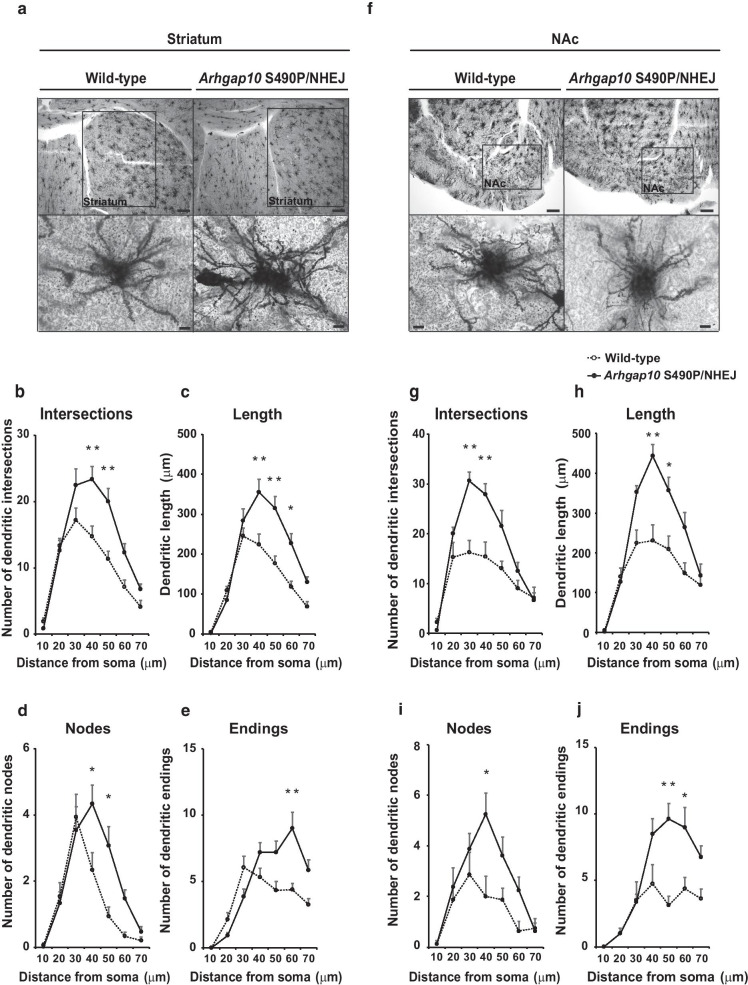
Abnormal neuronal morphology in the striatum and NAc of *Arhgap10* S490P/NHEJ mice. **a**, **f** Representative images of Golgi-stained single neurons in the striatum (**a**) and NAc (**f**). Scale bar: upper panel 300 μm and lower panel 10 μm. **b**–**e**, **g**–**j** quantitative analyses of **b**, **g** intersection, **c**, **h** length, **d**, **i** nodes, **e**, **j** ending in the **b**–**e**) striatum and **g**–**j** NAc. *p < 0.05, **p < 0.01. All data are expressed as means ± SEM (n = 8–15 neurons from 4–5 mice in each genotype)

It is well known that RhoA and Cdc42 signaling is involved in the regulation of neuronal morphology [32]. To test the possible changes in neuronal morphology in the striatum and NAc of *Arhgap10* S490P/NHEJ mice, the brains were subjected to Golgi staining (Fig. 3). Branching density and complexity of the neurons were assessed using a Sholl analysis method. The analysis revealed abnormal increases in intersections (Fig. 3a, b), length (Fig. 3a, c), nodes (Fig. 3a, d), and endings (Fig. 3a, e) in the striatal neurons of *Arhgap10* S490P/NHEJ mice. Similar results were observed in the accumbal neurons of *Arhgap10* S490P/NHEJ mice in intersections (Fig. 3f, g), length (Fig. 3f, h), nodes (Fig. 3f, i), and endings (Fig. 3f, j). These results suggested that the *Arhgap10* gene mutations led to an abnormal increase in neuronal complexity in neurons of the striatum and NAc.

### Changes in spine density and morphology in the striatum and NAc of *Arhgap10* S490P/NHEJ mice

**Fig. 4:**
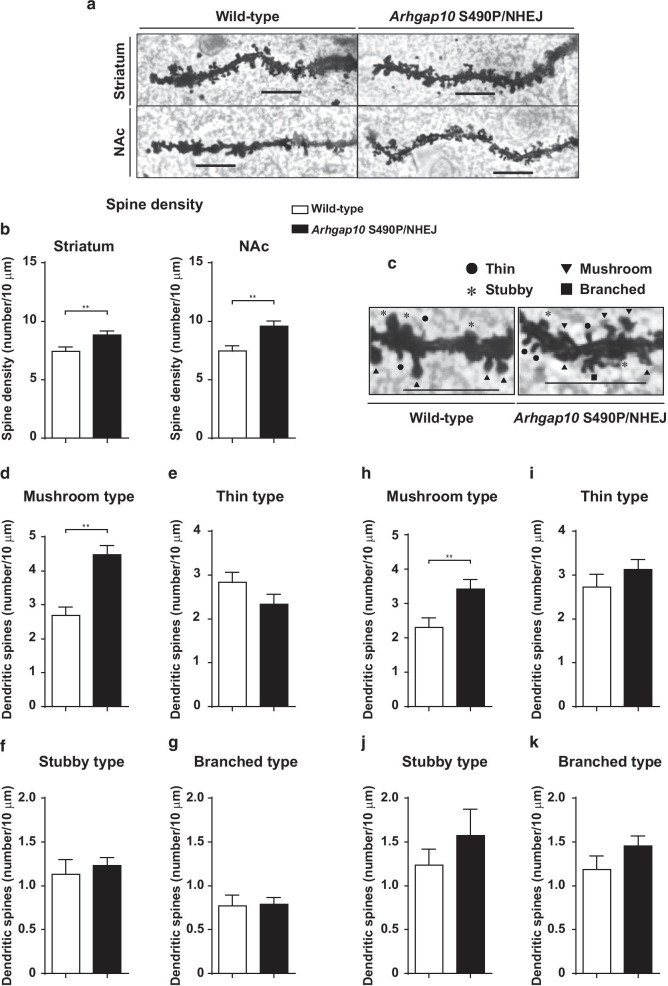
Changes in spine density and morphology in the striatum and NAc of *Arhgap10* S490P/NHEJ mice. **a** Representative images of the striatum and NAc dendric spines (Scale bar indicates 10 μm). **b** Spine densities of the striatum and NAc neurons. **c** Representative images of a dendritic segment illustrating different spine subtypes in NAc (Scale bar indicates 10 μm). **d**–**k** Quantification of dendritic spine: **d**, **h** mushroom, **e**, **i** thin, **f**, **j** stubby, and **g**, **k** brunched types in the **d**–**g** striatum and **h**–**k** NAc neurons, respectively. **p < 0.01. All data are expressed as means ± SEM (n = 16 neurons from 4 mice in each genotype)

Rho signaling is important for actin remodeling [33–35]. Stabilization of the actin cytoskeleton is essential for spine shape and density [34, 36, 37]. Spine cytoskeletal stabilization is altered in multiple cortical areas in schizophrenia [19]. Therefore, the number of spines on secondary dendrites was counted in the striatum and NAc of wild-type and *Arhgap10* S490P/NHEJ mice. Figure 4a shows representative images of dendrites in the striatum and NAc. The spine density was significantly increased in the striatal and accumbal neurons of *Arhgap10* S490P/NHEJ mice compared with wild-type mice (Fig. 4b).

Dendritic spines are often grouped based on their morphologies [38]. Spines were classified into four types (thin, stubby, branch, and mushroom, Fig. 4c). Mushroom spines, defined by their characteristically large head and narrow neck, contain the largest excitatory synapses, whereas thin spines are smaller, lack the large head and thin neck, and contain smaller excitatory synapses [39–41]. The numbers of mushroom-type spines were significantly increased in the striatum and NAc of *Arhgap10* S490P/NHEJ mice compared with wild-type mice (Fig. 4d, h). However, there were no significant differences in the thin, stubby, and branched types of spines between these two groups of mice (Fig. 4e–g). Similar results were observed in the NAc of *Arhgap10* S490P/NHEJ mice (Fig. 4i–k). These results suggested that *Arhgap10* gene mutations result in dendritic spine changes in the striatum and NAc, which may lead to activity-dependent neuronal dysfunction.

### Impairment of visual discrimination in *Arhgap10* S490P/NHEJ mice induced by treatment with methamphetamine

**Fig. 5:**
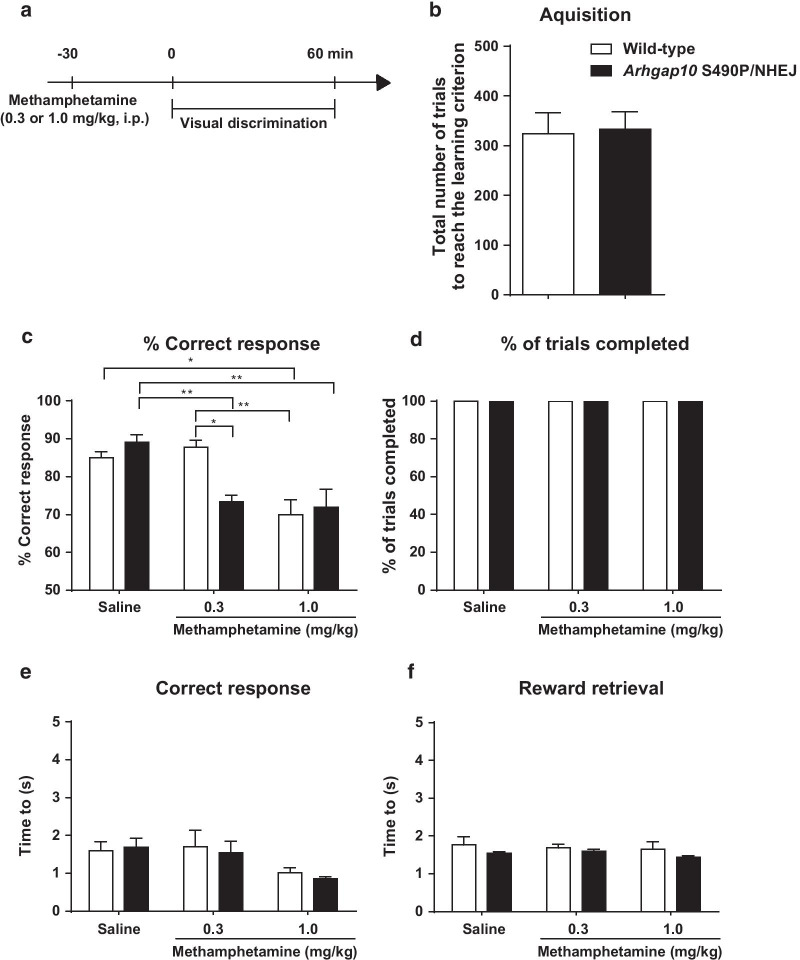
Impairment of visual discrimination in *Arhgap10* S490P/NHEJ mice induced by methamphetamine. **a** Mice were administered methamphetamine (0.3 or 1.0 mg/kg. i.p.) 30 min before the visual discrimination task. **b** The total number of trials to reach the learning criterion in the visual discrimination task. **c** Percentage of the correct response. **d** Percentage of trials completed. **e** Time to correct response. **f** Time to reward retrieval. *p < 0.05, **p < 0.01. All data are expressed as means ± SEM (wild-type mice n = 6, *Arhgap10* S490P/NHEJ mice n = 7)

The schizophrenia patient (Case #5) who has both an exonic deletion and a missense variant (p.S490P) in *Arhgap10* showed visual hallucination and cognitive impairment [13]. Thus, we analyzed the cognitive function of *Arhgap10* S490P/NHEJ mice using the visual discrimination task (Fig. 5a), in which normal performance depends on the intact function of the cortico-striato-thalamic circuitry [42]. Moreover, since we have previously demonstrated that an increase in neuronal activity in the striatum is associated with an impairment of performance in the visual discrimination task [26], the functional consequences of morphological abnormality of neurons in the striatum and NAc of *Arhgap10* S490P/NHEJ mice may be detected by the task. Somewhat unexpectedly, no differences were observed in the total number of trials to reach the learning criterion of the visual discrimination task (Fig. 5b) as well as reversal learning (Additional file 2: Figure S1) between wild-type and *Arhgap10* S490P/NHEJ mice, suggesting that the two groups of mice have the comparable ability in the visual discrimination learning and reversal learning.

Methamphetamine has been shown to induce cognitive impairment in the visual discrimination task [43]. Furthermore, the methamphetamine-induced hyperlocomotion was significantly potentiated in *Arhgap10* S490P/NHEJ mice compared with wild-type mice [13]. Therefore, the effect of methamphetamine on the performance in *Arhgap10* S490P/NHEJ mice was tested using the visual discrimination task. Methamphetamine (0.3 or 1.0 mg/kg) was administered 30 min before the test once mice had exhibited stable discrimination performance (more than 80% correct responses) for 2 consecutive days. Methamphetamine (1.0 mg/kg) significantly reduced the accuracy in the visual discrimination test in both wild-type and *Arhgap10* S490P/NHEJ mice. Of note, at a lower dose of 0.3 mg/kg, methamphetamine significantly reduced the percentage of correct responses in *Arhgap10* S490P/NHEJ mice but not in wild-type mice (Fig. 5c). The reduction of correct responses was not due to motor dysfunction or motivational change because all three groups of mice completed the 30 trials within 1 h (Fig. 5d), and there were no differences between groups in time to make correct responses (Fig. 5e) or retrieve reward (Fig. 5f). These results suggested that *Arhgap10* S490P/NHEJ mice are more vulnerable to methamphetamine treatment in terms of cognitive function than wild-type mice.

### Methamphetamine-induced changes in neuronal activity of the dorsal striatum and NAc in *Arhgap10* S490P/NHEJ mice

Methamphetamine increases DA release from the nerve terminals of dopaminergic neurons that project from the ventral tegmental area to the NAc [44, 45]. A recent study indicated that acute amphetamine treatment upregulates RhoA activity and dopamine transporter internalization [46]. One possible explanation for the higher sensitivity of *Arhgap10* S490P/NHEJ mice to methamphetamine-induced cognitive impairment in the visual discrimination task as well as methamphetamine-induced hyperlocomotion [13], is that methamphetamine-induced DA release may be potentiated in *Arhgap10* S490P/NHEJ mice compared with wild-type mice. To test this possibility, the extracellular DA levels in the NAc were measured using an in vivo microdialysis method. As shown in Additional file 3: Figure S2, no differences in the basal level as well as methamphetamine (1.0 mg/kg)-induced DA release were observed between wild-type and *Arhgap10* S490P/NHEJ mice. These findings suggest that functional changes in presynaptic DA neurons in *Arhgap10* S490P/NHEJ mice are minimal and may not play a significant role in the higher sensitivity to methamphetamine in *Arhgap10* S490P/NHEJ mice.

**Fig. 6:**
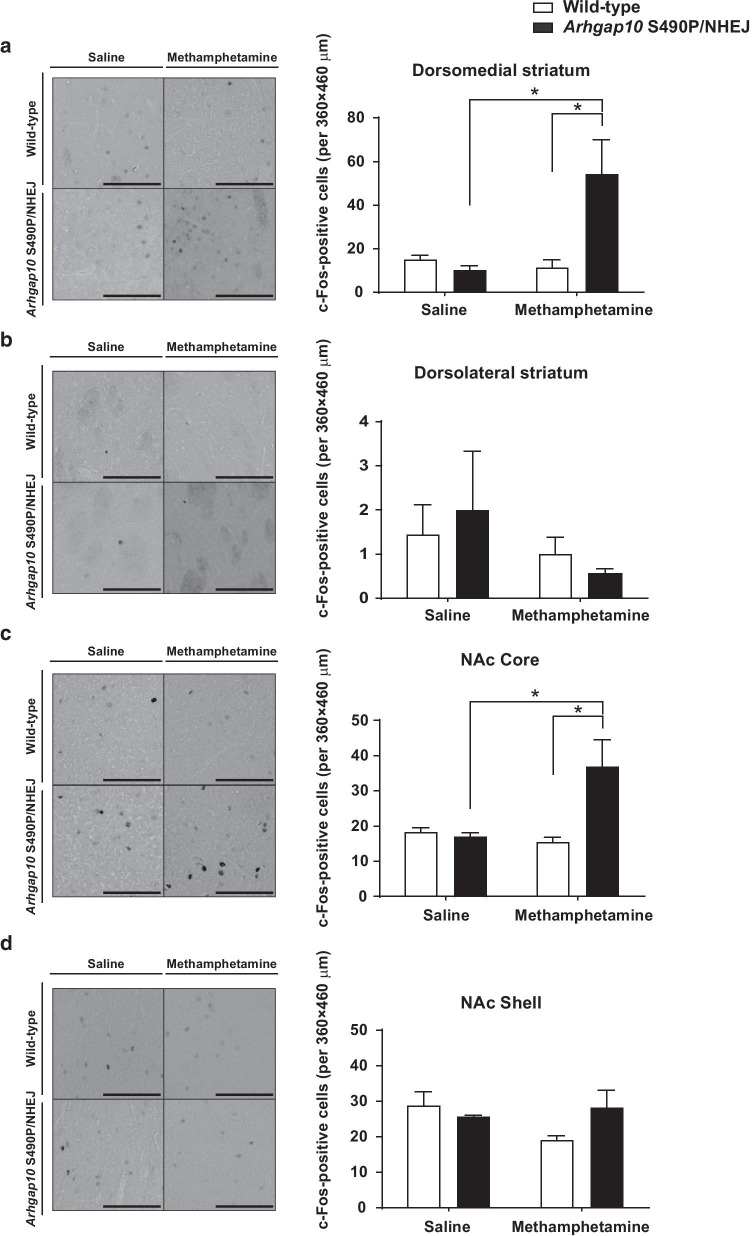
Methamphetamine-induced changes in neuronal activity of the dorsal striatum and NAc in *Arhgap10* S490P/NHEJ mice. **a**–**d** Representative images and the number of c-Fos-positive cells in the (**a**) dorsomedial striatum, (**b**) dorsolateral striatum, (**c**) NAc core, and (**d**) NAc shell. Scale bar indicates 100 μm. *p < 0.05. All data are expressed as means ± SEM (n = 9 slices from 3 mice in each group)

Neurons in the striatum and NAc of *Arhgap10* S490P/NHEJ mice exhibit more complex morphology (Fig. 3) and higher spine density including mushroom-type mature spines (Fig. 4) compared with those in wild-type mice. Accordingly, these postsynaptic changes in the striatum and NAc may be related to the higher sensitivity and enhanced response to methamphetamine in *Arhgap10* S490P/NHEJ mice. To test our hypothesis, the number of c-Fos-positive cells was compared in the dorsomedial and dorsolateral striatum, NAc core, and NAc shell between *Arhgap10* S490P/NHEJ and wild-type mice following methamphetamine (0.3 mg/kg) treatment. Quantitative analyses revealed that methamphetamine (0.3 mg/kg) significantly increased the number of c-Fos-positive cells in the dorsomedial striatum (Fig. 6a) and NAc core (Fig. 6c) in *Arhgap10* S490P/NHEJ mice but not wild-type mice. No significant differences were observed in the dorsolateral striatum (Fig. 6b) and NAc shell (Fig. 6d) between methamphetamine-treated wild-type and *Arhgap10* S490P/NHEJ mice. Taken together, the higher sensitivity and enhanced response to methamphetamine in *Arhgap10* S490P/NHEJ mice may be associated with the morphological abnormality of neurons in the dorsomedial striatum and NAc core.

## Discussion

In the present study, we demonstrated that *Arhgap10* gene expression increases postnatally in an age-dependent manner in the mouse brain. Especially in adulthood, a high level of *Arhgap10* gene expression was found in the striatum and NAc. It is well known that 95% of neurons within the striatum and NAc are medium spiny neurons (MSNs), which are a special type of GABAergic inhibitory cells [47, 48]. MSNs are subdivided into 2 classes; dopamine D1 receptor (D1R)-expressing MSNs (D1R-MSNs) and dopamine D2 receptor (D2R)-expressing MSNs (D2R-MSNs [49]. A previous study using single-cell RNA sequencing of mouse striatum revealed that *Arhgap10* gene is expressed in various cell types including D1R-MSNs, D2R-MSNs, pericytes, and astrocytes [50]. We also found high expression of *Arhgap10* gene in the cerebellum, but its functional significance remains obscure.

ARHGAP10 participates in the regulation of RhoA and Cdc42 activity [20]. Deletions of RhoA GAP genes, such as *Oligophrenin1* and *p190RhoGAP*, led to an increase in RhoA activity, whereas deletion of the *SNX26*, which belongs to Cdc42 GAP family, resulted in activation of Cdc42 signal [51–53]. In vitro experiments have already demonstrated that the *Arhgap10* S490P mutation increases RhoA activity due to a resultant decrease in the interaction of ARHGAP10 with GTP-RhoA [13]. In this study, we observed that not only phospho-MYPT1 levels but also phospho-PAK1/2 levels were upregulated in the striatum and NAc of *Arhgap10* S490P/NHEJ mice. These findings suggested that RhoA and Cdc42 signaling was promoted in the striatum and NAc of *Arhgap10* S490P/NHEJ mice, namely the *Arhgap10* gene mutations may be accelerating RhoA and Cdc42 signals in the striatum and NAc.

Postmortem studies revealed that the spine density of cortical parts is decreased in schizophrenia patients [54, 55], whereas dendritic synaptic density in the striatum is increased [56]. Similarly, a significant decrease in spine density in the medial prefrontal cortex of *Arhgap10* S490P/NHEJ mice was reported in our previous study [13], whereas a significant increase in spine density, as well as neuronal complexity in the striatum and NAc, was demonstrated in the present study. Rho signaling controls neuronal branching pattern and dendritic spine density [57–61]. Constitutive active RhoA decreases spine density, whereas activation of Cdc42 increases spine density [53, 57, 59, 60]. These findings suggested that the contribution of Rho signals regulated by ARHGAP10 is different in the medial prefrontal cortex and striatum. Further studies are required to conclude a causal association between the *Arhgap10* mutation and neuronal morphology in MSNs.

Changes in dendritic spine shape have been correlated with behavioral alterations, such as learning and memory, and may provide a structural basis for plasticity in the brain [62]. Structural changes in spines can occur within seconds and are coupled to changes in synaptic activity [63]. Previous reports indicated RhoA and Cdc42 activation in single dendritic spines undergoing structural plasticity associated with long-term potentiation [64, 65]. The dendritic spine shape is classified and synaptic ability is different among them [36, 39–41]. Mushroom-type spines represent a more mature population than other types of spines, and their large head/neck ratio is thought to contribute to their function as stable excitatory synapses [63]. Of note, repetitive amphetamine or methamphetamine treatment induces behavioral sensitization in mice [66, 67], which is accompanied by an increase in mushroom-type spine density in MSNs [68, 69]. In the present study, we found an increase in mushroom-type spine density in the striatal and accumbal neurons of *Arhgap10* S490P/NHEJ mice that have a higher sensitivity to methamphetamine. These results suggest that morphological abnormalities in the striatum and NAc of *Arhgap10* S490P/NHEJ mice may be related to their hypersensitivity to methamphetamine. MSNs in the striatum and NAc receive dopaminergic input from the substantia nigra/ventral tegmental area, as well as excitatory glutamatergic input from the cortex/thalamus [70]. In the present study, an in vivo microdialysis study showed that basal and methamphetamine-induced DA release in the NAc of *Arhgap10* S490P/NHEJ mice were comparable to those in wild-type mice, indicating that the function of mesostriatal DA pathway is normal in *Arhgap10* S490P/NHEJ mice.

The touchscreen-based visual discrimination task can be used to assess aspects of associative learning and perceptual ability in rodents [28], and visual perception and recognition are impaired in schizophrenia patients [71]. Brigman et al. (2013) reported that the principal brain regions are activated during choice behavior. An excitotoxic lesion as well as in vivo neuronal recording suggested that normal performance in the visual discrimination task depends on the intact function of the cortico-striato-thalamic circuitry. The neuronal circuit for this type of learning behavior is preserved in humans, non-human primates, and rodents [72]. *Arhgap10* S490P/NHEJ mice showed normal performance in the visual discrimination and reversal learning task, indicating that learning ability may be normal in *Arhgap10* S490P/NHEJ mice. Nevertheless, a low dose of methamphetamine impaired visual discrimination performance in *Arhgap10* S490P/NHEJ mice but not wild-type mice, although there was no difference in methamphetamine-induced DA release between the two groups. Furthermore, *Arhgap10* S490P/NHEJ mice have an abnormality in the morphology of striatal MSNs with a higher density of mushroom-type mature spines compared with wild-type mice. Thus, the MSNs in the striatum/NAc of *Arhgap10* S490P/NHEJ mice may exhibit abnormal responses upon DA receptor stimulation after methamphetamine administration. This hypothesis was consistent with clinical findings that schizophrenia patients are sensitive to methamphetamine [73].

In conclusion, our results suggested that *Arhgap10* gene mutations have a pathophysiologic and pathogenic role, involving structural and functional changes in the MSNs of striatum and NAc, in schizophrenia. Targeting the regulation of Rho GTPase and the downstream signaling may provide new therapeutic approaches for the treatment of schizophrenia patients with *Arhgap10* gene mutations.

## Supplementary Information

**Additional file 1 **Table S1. Summary of the statistical analyses used in this study

**Additional file 2: **Figure S1. Performance of *Arhgap10* S490P/NHEJ mice in the visual discrimination reversal learning task. (a) Percentage of correct responses in reversal learning. (b) Total trials to reversal learning criteria. All data are expressed as means ± SEM (wild-type mice n = 4, *Arhgap10* S490P/NHEJ mice n = 5).

**Additional file 3: **Figure S2. Methamphetamine-induced DA release in the NAc of *Arhgap10* S490P/NHEJ mice. (a) Basal extracellular levels of DA and (b) methamphetamine-induced (1 mg/kg, i.p.) DA release in the NA of *Arhgap10* S490P/NHEJ mice were determined using a microdialysis method. Each fraction was collected for 5 min. All data are expressed as means ± SEM (n = 6 mice in each genotype).

## Data Availability

All data generated or analyzed during this study are included in this published article. Additional inquiries can be directed to the corresponding author.

## Abbreviations

ANOVA: Analysis of variance
*Arhgap10*: Rho GTPase activating protein 10
CNVs: Copy-number variations
D1R: Dopamine D1 receptor
D2R: Dopamine D2 receptors
DA: Dopamine
i.p.: Intraperitoneal
PAKs: P21-activated kinases
PBS: Phosphate-buffered saline
qPCR: Quantitative real-time polymerase chain reaction
MSNs: Medium spiny neurons
MYPT1: Myosin phosphatase-targeting subunit 1
NAc: Nucleus accumbens
NHEJ: Non-homologous end-joining
