# Supplementary figures and images for: Mice carrying a schizophrenia-associated mutation of the Arhgap10 gene are vulnerable to the effects of methamphetamine treatment on cognitive function: association with morphological abnormalities in striatal neurons
Source: Mol Brain. 2021 Jan 22;14:21. doi: 10.1186/s13041-021-00735-4 (PMC7821731; doi:10.1186/s13041-021-00735-4)

Figure S1.

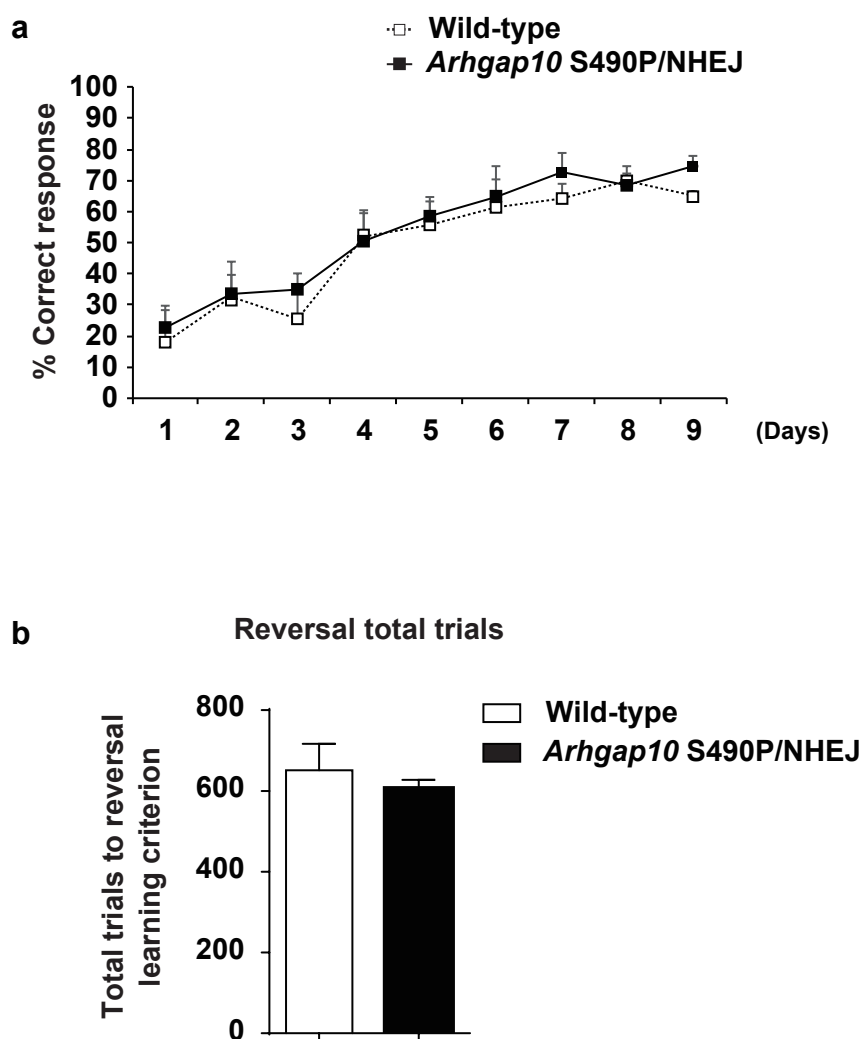

Supplement: Supplementary file 2 — Additional file 2: Figure S1. Performance of Arhgap10 S490P/NHEJ mice in the visual discrimination reversal learning task. (a) Percentage of correct responses in reversal learning. (b) Total trials to reversal learning criteria. All data are expressed as means ± SEM (wild-type mice n = 4, Arhgap10 S490P/NHEJ mice n = 5). [file 13041_2021_735_MOESM2_ESM.pdf]

Figure S2.

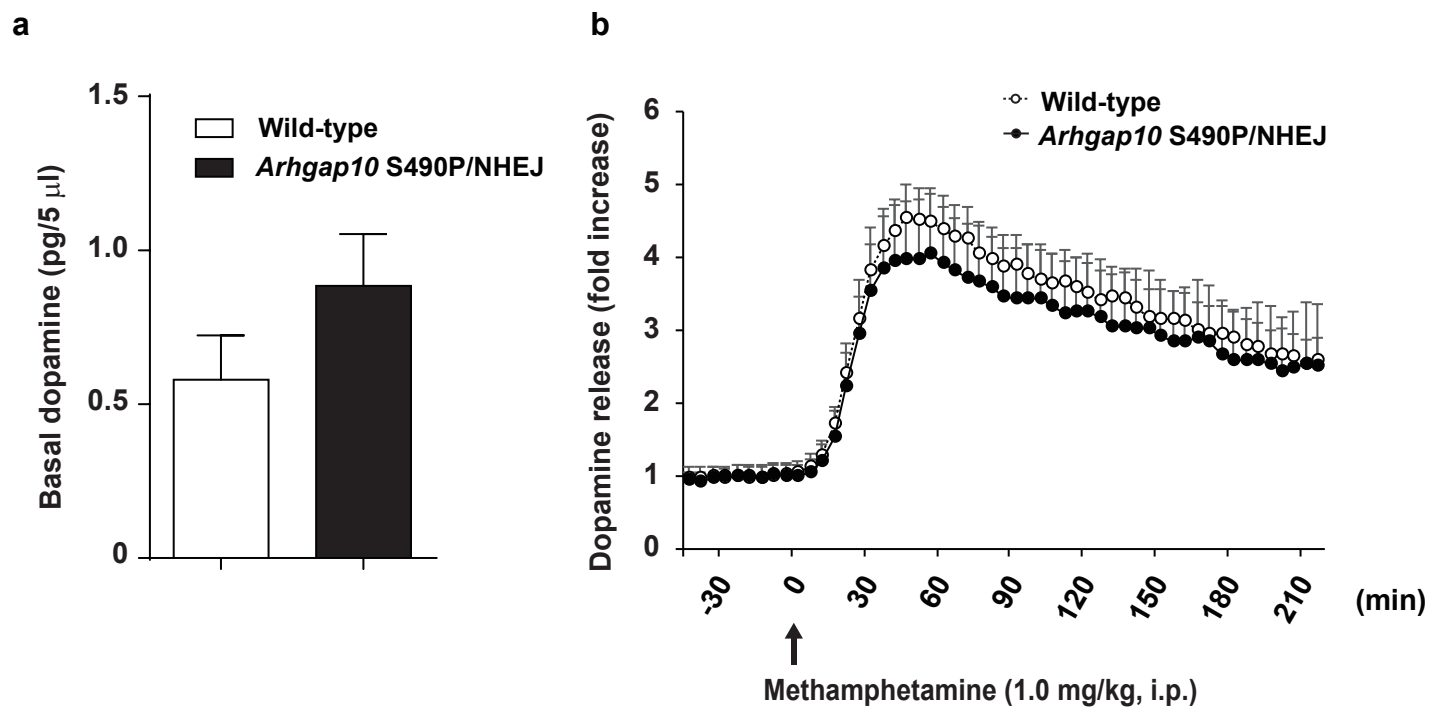

Supplement: Supplementary file 3 — Additional file 3: Figure S2. Methamphetamine-induced DA release in the NAc of Arhgap10 S490P/NHEJ mice. (a) Basal extracellular levels of DA and (b) methamphetamine-induced (1 mg/kg, i.p.) DA release in the NA of Arhgap10 S490P/NHEJ mice were determined using a microdialysis method. Each fraction was collected for 5 min. All data are expressed as means ± SEM (n = 6 mice in each genotype). [file 13041_2021_735_MOESM3_ESM.pdf]
